# Author Correction: Measuring sensitivity to social distancing behavior during the COVID-19 pandemic

**DOI:** 10.1038/s41598-023-29283-8

**Published:** 2023-02-07

**Authors:** Constantine E. Kontokosta, Boyeong Hong, Bartosz J. Bonczak

**Affiliations:** 1 Marron Institute of Urban Management, New York University, 370 Jay Street, Brooklyn, 11201 NY USA; 2 Center for Urban Science and Progress, New York University, 370 Jay Street, Brooklyn, 11201 NY USA

Correction to: *Scientific Reports* 10.1038/s41598-022-20198-4, published online 29 September 2022

The original version of this Article contained an error in the Funding section.

“This material is based upon work supported by the National Science Foundation under awards No. 1926470 and No. 2028687. Any opinions, findings, and conclusions expressed in this paper are those of the authors and do not necessarily reflect the views of any supporting institution. All errors remain the authors.”

now reads:

“This material is based upon work supported by the National Science Foundation under awards No. 1926470, No. 2028687, and No. IIS-2040898. Any opinions, findings, and conclusions expressed in this paper are those of the authors and do not necessarily reflect the views of any supporting institution. All errors remain the authors.”

The original Article has been corrected.

